# Digitization of Measurement-Based Care Pathways in Mental Health Through REDCap and Electronic Health Record Integration: Development and Usability Study

**DOI:** 10.2196/25656

**Published:** 2021-05-20

**Authors:** Steve Hawley, Joanna Yu, Nikola Bogetic, Natalia Potapova, Chris Wakefield, Mike Thompson, Stefan Kloiber, Sean Hill, Damian Jankowicz, David Rotenberg

**Affiliations:** 1 Krembil Centre for Neuroinformatics Centre for Addiction and Mental Health Toronto, ON Canada; 2 Clinical Applications Centre for Addiction and Mental Health Toronto, ON Canada; 3 General Adult Psychiatry and Health Systems Division Centre for Addiction and Mental Health Toronto, ON Canada

**Keywords:** REDCap, electronic health record, systems integration, measurement-based care, hospital information systems

## Abstract

**Background:**

The delivery of standardized self-report assessments is essential for measurement-based care in mental health. Paper-based methods of measurement-based care data collection may result in transcription errors, missing data, and other data quality issues when entered into patient electronic health records (EHRs).

**Objective:**

This study aims to help address these issues by using a dedicated instance of REDCap (Research Electronic Data Capture; Vanderbilt University)—a free, widely used electronic data capture platform—that was established to enable the deployment of digitized self-assessments in clinical care pathways to inform clinical decision making.

**Methods:**

REDCap was integrated with the primary clinical information system to facilitate the real-time transfer of discrete data and PDF reports from REDCap into the EHR. Both technical and administrative components were required for complete implementation. A technology acceptance survey was also administered to capture physicians’ and clinicians’ attitudes toward the new system.

**Results:**

The integration of REDCap with the EHR transitioned clinical workflows from paper-based methods of data collection to electronic data collection. This resulted in significant time savings, improved data quality, and valuable real-time information delivery. The digitization of self-report assessments at each appointment contributed to the clinic-wide implementation of the major depressive disorder integrated care pathway. This digital transformation facilitated a 4-fold increase in the physician adoption of this integrated care pathway workflow and a 3-fold increase in patient enrollment, resulting in an overall significant increase in major depressive disorder integrated care pathway capacity. Physicians’ and clinicians’ attitudes were overall positive, with almost all respondents agreeing that the system was useful to their work.

**Conclusions:**

REDCap provided an intuitive patient interface for collecting self-report measures and accessing results in real time to inform clinical decisions and an extensible backend for system integration. The approach scaled effectively and expanded to high-impact clinics throughout the hospital, allowing for the broad deployment of complex workflows and standardized assessments, which led to the accumulation of harmonized data across clinics and care pathways. REDCap is a flexible tool that can be effectively leveraged to facilitate the automatic transfer of self-report data to the EHR; however, thoughtful governance is required to complement the technical implementation to ensure that data standardization, data quality, patient safety, and privacy are maintained.

## Introduction

### Background

A key component of measurement-based care (MBC) in mental health relies on the ability of patients to complete self-assessments. These assessments can be used in conjunction with evidence-informed practice guidelines to monitor patient progress and inform clinical decisions for patient-centered care plans. MBC can improve outcomes by rapidly detecting patients with deteriorating symptoms and by effectively quantifying persistent symptoms [1]. The Centre for Addiction and Mental Health (CAMH) is the largest mental health hospital in Canada, supporting over 34,000 patients per year across diverse clinical programs. CAMH has developed and deployed a number of integrated care pathways (ICPs), which include MBC as the main, evidence-based aspect of care. Broadly used across many clinical domains, ICPs are structured care plans that follow evidence-based guidelines to improve patient outcomes [2] and may include definitions for specific medications or treatment strategies [3,4].

In addition to providing clinical care, CAMH is Canada’s leading mental illness research facility. In recent years, REDCap (Research Electronic Data Capture; Vanderbilt University) has been instrumental to this research effort [5]. REDCap is a secure, Health Insurance Portability and Accountability Act–compliant, web-based application for building and managing electronic surveys and assessments [6]. Developed in 2004 by researchers at Vanderbilt University, REDCap was designed to provide a straightforward means of data management for the local research community [7]. Since its inception, the platform has evolved through several phases, first through select development partnerships and then through a growing international consortium that currently represents a diverse global community spanning over 3600 institutions in more than 130 countries with more than a million users worldwide [8]. The platform is available to academic, nonprofit, and government organizations through a no-cost consortium model, whereby partner institutions are provided the source code and installation files for REDCap after agreeing to the end user license agreements terms outlined by Vanderbilt University.

REDCap was launched at CAMH in 2015 for research data collection and operational support. Since then, the platform has grown to more than 1000 users who have created over 1800 projects culminating in more than 200,000 individual records. In 2017, a second, fully validated instance of REDCap was launched to support data collection for Health Canada regulated clinical trials. In 2018, because of the growing familiarity with REDCap at CAMH and knowledge of its flexibility, customizability, and community support, a demand for a version of REDCap to support clinical data began to emerge. Specifically, this version of REDCap would be used for ICP delivery. It was posited that REDCap’s flexibility and its ability to manage complex workflows would significantly improve the previously paper-based methods used for ICP delivery. Importantly, however, this version of REDCap would need to be integrated with the CAMH electronic health record (EHR), a CAMH-branded version of Cerner Millennium called I-CARE.

CAMH achieved the Healthcare Information Management Systems Society (HIMSS) Adoption Model for Analytics Maturity Stage 6 in 2018 and the Electronic Medical Record Adoption Model Stage 7 in 2017 [9], indicating excellent hospital-wide adoption and compliance. Electronic data capture is used throughout the hospital and coordinated as the *sole source of truth* legal health records. To enhance the flexibility of electronic data collection for MBC, a *clinical* instance of REDCap was launched and connected to Cerner via a systems integration layer to facilitate the automatic transfer of completed assessments to the patient chart in both PDF and discrete formats. This integration extends capabilities for complex survey queues and branching assessments as needed by clinical pathways while maintaining the integrity of the health record.

### Objectives

In this development and usability study, we describe the technical implementation and governance model of a clinical instance of REDCap to support digital MBC in multiple clinical environments. We detail the technical requirements for real-time transfer of self-assessments collected in REDCap to the patient’s chart in Cerner in both PDF and discrete variable formats. In addition, we describe the clinical governance structure necessary to sustain high-quality clinical data and support standardized implementation across clinics. A pertinent clinical use case is outlined to illustrate the utility and applicability of REDCap to support scalable and extensible structured mental health MBC at CAMH and beyond.

## Methods

### Overview

Evaluation of the ICPs at CAMH identified a need to deploy complex bundles of assessments over time that could be dynamically adjusted based on indicators such as severity or frequency of symptoms. The functionality of REDCap is well suited to meet these requirements. By delivering standardized assessments longitudinally, modified through branching and survey queue logic as appropriate, necessary psychological dimensions could be sampled to inform physician decision making throughout the course of care. The clinical instance of REDCap (Clinical REDCap) was the third installment of REDCap at CAMH, preceded by a research instance and a fully validated instance for regulated clinical trials. REDCap version 9.1 was used for this implementation.

### Technical Implementation

The technical implementation to integrate REDCap with Cerner included (1) tablet deployment in the clinic; (2) automated transfer of completed self-assessments in a PDF format to the patient chart; and (3) automated transfer of completed self-assessments in a discrete format to the patient chart for trending, analysis, and more complex physician decision support.

#### Tablet Deployment and Automated Survey Retrieval Tool

Samsung Galaxy Tab Android tablets were provided to clinical assistants to facilitate patient self-assessment data entry into REDCap. As a technical safeguard, a kiosk app (Kioware) was configured to limit tablet use to preapproved apps and webpages, in this case, Google Chrome and REDCap, respectively.

**Figure 1:**
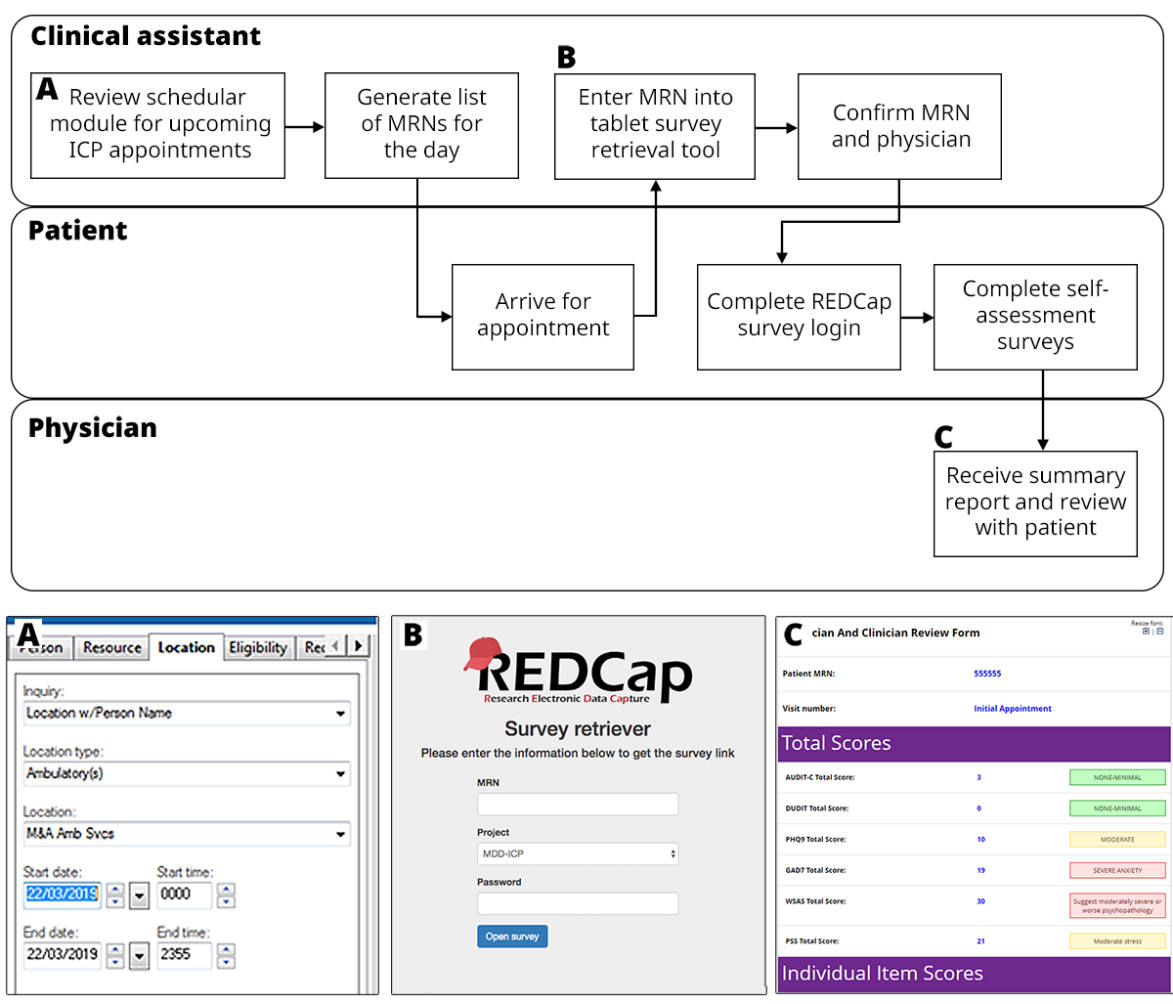
Clinical REDCap workflow and example screens for a typical follow-up appointment. (A) Cerner scheduler view. (B) Custom survey retriever tool used as a tablet homepage. (C) Summary report built as a REDCap survey. ICP: integrated care pathways; MRN: medical record number; REDCap: Research Electronic Data Capture.

In collaboration with the clinic managers and physician teams, work was undertaken to streamline the administration of tablets and surveys for patients and staff. To simplify the clinical workflow (Figure 1), a custom *survey retriever* web app was developed using the REDCap application programming interface (API). This tool accepts the patient medical record number (MRN) and ICP name and retrieves the unique survey queue link from the *next available* set of self-assessments in the care pathway for a particular patient. Using this tool, the clinical assistant may load the appointment’s set of assessments, beginning with a confirmation survey asking the clinical assistant to confirm the MRN, appointment date, and the patient’s physician to ensure compliance and accuracy. The physician field on this confirmation form is populated using a dynamic SQL (Structured Query Language) field, which draws from a separate REDCap project that lists all ICP physician names or emails and their current status (eg, active or inactive). It allows clinical end users to easily update the physician list without needing to dynamically modify a dropdown list directly in the Online Designer. As a final patient verification step before data entry, the REDCap survey log-in feature is used to prompt the patient to enter a personal identification number (PIN) based on their date of birth.

#### Transfer of PDFs to Cerner

To ensure the integrity of the health record, individually completed self-assessments were automatically transmitted as PDFs to the patient’s EHR in Cerner and were filed in the *documents* section, specifically under *assessments and plans*. For convenience, an aggregate report is automatically generated within REDCap by piping values into a standalone REDCap survey emailed to the primary clinician or physician upon assessment package completion (Figure 1). These reports summarize patient response data onto a single page; however, they are not sent to Cerner, as the data have already been transferred as individual files.

As the patient completes assessments, a REDCap data entry trigger (DET) delivers the project ID, record ID, event name, instrument, and form status to a custom-built data export web service. The service listens for DETs and sends a request back to REDCap, thus providing real-time data export functionality. Following the DET, the service makes a request to REDCap using the API method *export PDF file of data collection instruments* to retrieve a PDF document of the completed assessment (eg, the Patient Health Questionnaire-9 [PHQ-9]), and the document is written into a securely shared directory. Each PDF is given a unique file name that follows a predefined convention, as follows:

[mrn]_[encounter]_[assessment_code]_[date]_[time].pdf

**Figure 2:**
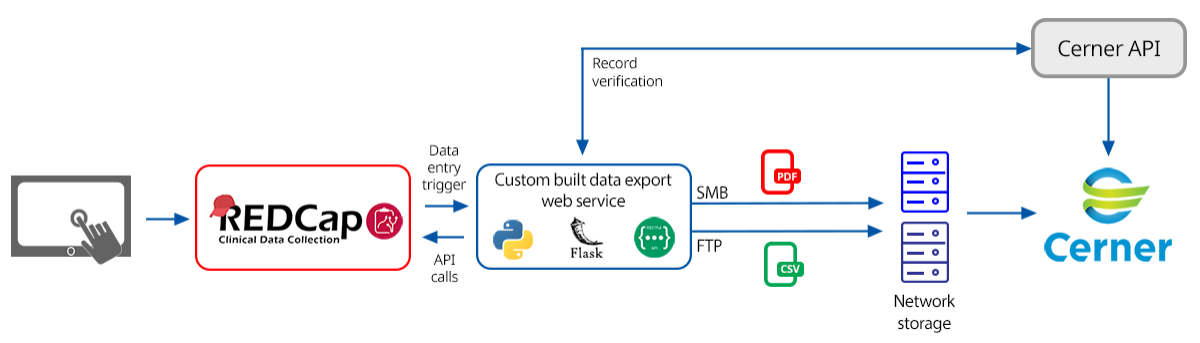
Clinical REDCap workflow and example screens for a typical follow-up appointment for a patient in the major depressive disorder integrated care pathway. API: application programming interface; FTP: file transfer protocol; REDCap: Research Electronic Data Capture; SMB: server message block.

This process ensures further compatibility with the Cerner interface, which uses a purpose-built Cerner AXRM COLD Feed (Cerner) [10] to import the document into the patient chart (Figure 2).

#### Transfer of Discrete Data to Cerner

Similar to PDF export from REDCap, the transfer of discrete values also uses DETs and the REDCap API. In this case, the *export records* API method was used. This method returns the values of the completed assessment fields (eg, PHQ-9) and system variables, such as timestamps. Once the discrete data are retrieved, the web service executes the code to format this information as a comma-separated values (CSV) file that follows a specified convention, as follows: [timestamp].csv for the name and [mrn],[encounter],[uid],[assessment_code],[timestamp], [assessment_field1],[assessment_field2],...,[assessment_fieldN] for CSV content.

UID is a unique identifier of a completed assessment within the REDCap instance generated using the project ID, record ID, instrument code, and visit code. This identifier ensures that the correct form is updated in Cerner if the data in REDCap are modified. At this step, assessment variables are also renamed to be correctly mapped to the corresponding Cerner fields. The CSV file is then written to a directory on a file transfer protocol server. This directory is periodically scanned by a custom Cerner interface, which converts the CSV data into an HL7 unsolicited results (ORU) message. The interface then routes the HL7 to the standard Cerner external systems inbound servers for processing and passes the values into the results view of the chart, where the data are displayed in a table format. Currently, the interface only accepts numerical results, but future updates may include the processing of REDCap data sent in JavaScript Object Notation (JSON) to allow greater flexibility for data import.

#### Record Verification and Linkage

The primary verification mechanism for data transfer relies on correctly entering the MRN and a Cerner-specific appointment identifier called the *Encounter* number into REDCap. A custom Cerner API (Figure 2) was built, which accepts these parameters, checks whether these values are valid and belong to the same patient, and returns a JSON-formatted response. Upon submitting a data entry form in REDCap, a DET initializes a custom-built data export web service that sends the MRN and *Encounter* number to the custom Cerner API. If all the criteria are met and a successful response is received, the web service proceeds to create the document and discrete files. If the criteria are not met, the transfer is terminated, and an error message is logged and sent via email to the study contact. The message can be one of the following: “Missing MRN,” “Missing Encounter,” or “MRN and Encounter mismatch.”

### Clinical REDCap Governance

To support technical development and integration, a robust and consistent governance model was necessary to ensure compliance with the legal health record and maintain the standardization necessary to effectively support scalable and reproducible MBC. We outline the governance practices adopted by our institution in a format that can be applied to other similar hospitals.

#### Clinical Governance Structures

The Clinical REDCap governance structure and principles were codeveloped with clinical and privacy stakeholders to establish core operating constraints. The underlying theme of these principles establishes Clinical REDCap as an extension of normal EHR data collection capabilities and establishes that clinically relevant information must pass through the primary EHR to maintain a consistent health record. Changes to the care pathway within REDCap must be reviewed and approved by clinical governance committees, consistent with the review process for changes to Cerner. In general, institutional clinical governance committees are accountable for final decisions relating to platform governance and approval of forms used for clinical care; REDCap administrators are accountable for system administration and all project development, and the clinical applications (Cerner) team is accountable for final integration with the EHR. All parties were consulted or informed at various points where necessary.

**Figure 3:**
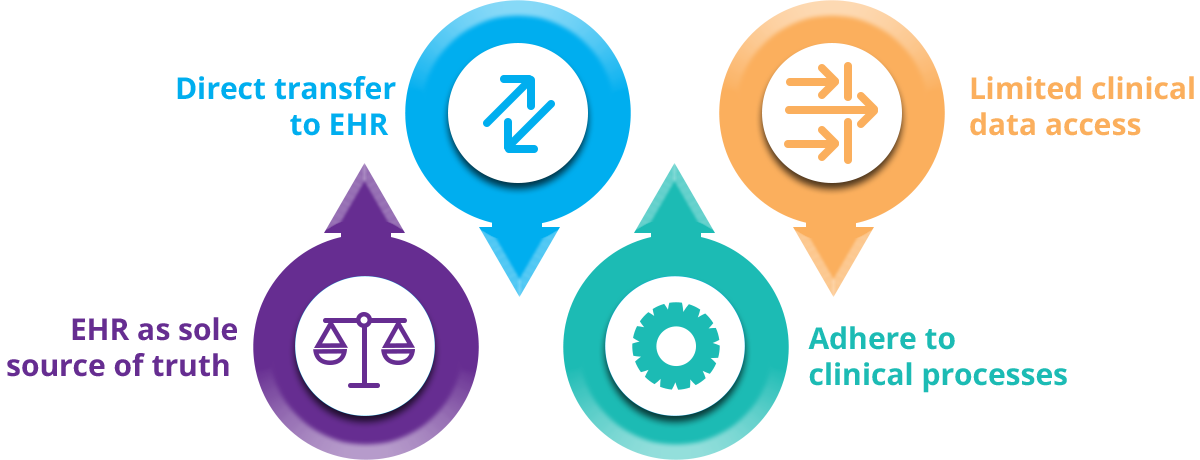
Four key principles of REDCap (Research Electronic Data Capture) integration with the Centre for Addiction and Mental Health EHR. EHR: electronic health record.

The collaboration between all stakeholders established a list of 4 key operating principles (Figure 3) that were used to inform all decisions relating to Clinical REDCap, as follows:

1. The hospital EHR will remain the *sole source of truth*.
2. Clinical data collected in REDCap will be transferred directly to the EHR.
3. Clinical REDCap will follow existing clinical measure or pathway approval processes.
4. Access to data collected through Clinical REDCap will follow existing processes pertaining to clinical records.

Although the specifics of principles 3 and 4 will differ depending on the institution and regional legislation, the high-level principle of clinical governance alignment is strongly recommended.

#### Controlled Access and Data Retention

Access to the clinical instance is limited at both the server and individual roles or permissions levels. The clinical instance sever is situated behind the CAMH firewall and is not accessible externally. At the application level, user management is tightly regulated under structured clinical roles established by privacy and health record experts to meet the needs of the clinical workflows and limit unnecessary data access.

Users of the platform fall into 2 broad categories: administrator and clinical (Textbox 1). *Administrators* are responsible for project creation, instrument design, and API configuration and are strictly limited to central REDCap operations staff. In the *clinical* category, 3 subcategories exist: clinical data lead, a clinical staff member who acts as the *owner* of a project and provides in-clinic training and basic support (eg, data corrections); clinical care provider, the role assumed by clinicians and physicians who may enter new records and clinical data; and clinical assistant, a support staff who retrieve survey access codes, load survey queues, and supply tablets to patients for self-assessment. Account provision and project access are handled solely by administrators. Each new user must complete mandatory training by reviewing the policies of Clinical REDCap, including the differences between clinical and research platforms, permitted user roles, and privacy considerations when using the system. User roles are assigned following a hierarchical process: users who request a clinical data lead role must have their request approved by the institutional clinical director. For subsequent clinical roles, account or project access is approved by the respective clinical data lead, who also determines the user role (clinical care provider or clinical assistant) for the project. This approval is required before the user account or project access is provided by an administrator. This hierarchical approval process conforms to the internal organizational structure for consistency and varies in other institutions; however, this approach provides a template for developing permissions modes that are sufficiently restrictive but allow secure access as necessary.

At present, Clinical REDCap project data reside in REDCap for a period aligned with institutional clinical data retention periods unless otherwise removed by a REDCap administrator by request of the clinical team and privacy office.

**Clinical REDCap (Research Electronic Data Capture) user roles and responsibilities.**

**Clinical REDCap administrator**

- Clinical REDCap staff responsible for account creation, modification, and suspension for all user types. Responsible for moving projects and draft changes to production. Limited to 2-3 staff members.

**Project developer administrator**

- Clinical REDCap staff responsible for creating REDCap projects and associated forms or surveys. Leads project validation to be carried out in collaboration with clinical stakeholders.

**Service administrator**

- Specialized account for managing secure services post data collection (eg, application programming interface data export to electronic health record).

**Clinical data lead**

- Clinical staff responsible for monitoring the integrity of the data, providing clinic-specific training to clinic users, supporting minor technical issues in the clinic, facilitating project validation, submitting project change requests, approving subordinate account requests, and serving as the main point of contact for developers and administrators.

**Clinical care provider**

- Clinical staff responsible for the administration and review of clinical assessments; can enter data that have been collected on paper into REDCap.

**Clinical assistant**

- Clinical support staff responsible for administering patient survey queue in the clinic and providing simple technical assistance.

#### Centralized Assessment and Project Development

**Figure 4:**
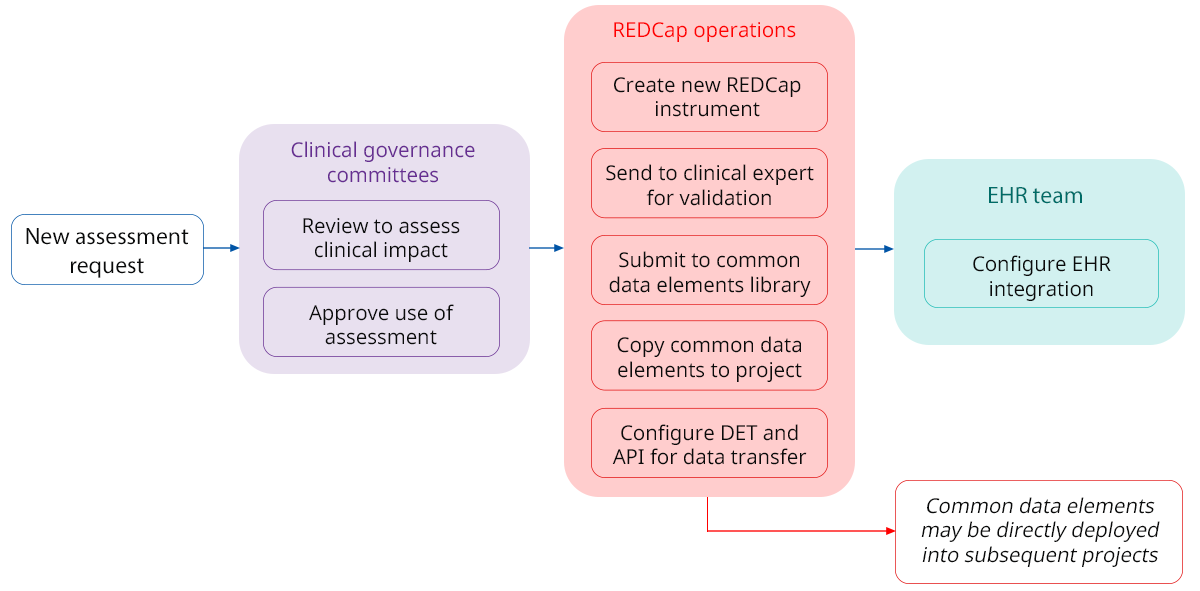
High-level workflow for clinical instrument development on the Clinical REDCap instance. The review is conducted by clinical committees to align assessments. API: application programming interface; DET: data entry trigger; EHR: electronic health record; REDCap: Research Electronic Data Capture.

All Clinical REDCap projects are built by a dedicated internal team of administrators that follow standardized naming and versioning practices for assessments and variables. All new assessments are clinically validated by CAMH clinicians or physicians and undergo strict approval processes through the hospital’s clinical data governance committees to ascertain alignment with existing clinical instruments and standard practice. Once validated, assessments are deployed into a *common data elements* library to support the rapid development of parallel and complementary pathways (Figure 4). The use of common data elements across all projects ensures clinical consistency and provides a standard set of elements that can be transmitted to the hospital EHR with little additional setup. Ancillary forms such as project or participant tracking, reporting, or summary forms are built ad hoc as requested on a project-by-project basis.

### Evaluation of Impact

The clinical impact was assessed by determining the number of physicians who adopted the ICP before and after implementing the Clinical REDCap project. In addition, the monthly enrollment of patients was determined by counting the number of patients enrolled in the paper-based process compared with the Clinical REDCap process on average per month after 6 months.

A short user acceptance survey was developed and sent to Clinical REDCap users in the clinical care provider role to assess clinicians’ and physicians’ attitudes toward Clinical REDCap. The survey drew questions from the technology acceptance model, which seeks to measure users’ perceived usefulness and perceived ease of use and how these factors may affect acceptance of new technology [11]. The survey consisted of 10 items measured on a 5-point Likert scale and included 3 free-text options for respondents to comment on their likes and dislikes and make suggestions for system improvement.

## Results

### Example Use Case: Major Depression Disorder Integrated Care Pathway

The major depression disorder integrated care pathway (MDD-ICP) provides a multidisciplinary and structured care management strategy, including interprofessional collaboration, standardized assessments, and evidence-based treatment algorithms. The pathway has been active at CAMH for 3 years, and a Clinical REDCap project was created as an alternative to paper-based data collection to digitize and support the clinic-wide implementation of the pathway in the fall of 2018. The data collected in Clinical REDCap (which are subsequently made available in the EHR) are used to support the MBC component of the MDD-ICP, which is used to inform treatment decisions.

### Data Entry Workflow

Figure 1 shows a typical Clinical REDCap workflow for patients in the MDD-ICP. Every morning, a clinical assistant reviews the EHR scheduler module to compile a list of patients scheduled for an ICP appointment. When a patient arrives for their appointment, the clinical assistant retrieves a tablet and enters the MRN into the survey retriever tool. Provided that a valid MRN (ie, an existing record in REDCap) is entered, the retriever tool will open the first survey of the next available follow-up event. In this case, the first survey is a confirmation page that asks the clinical assistant to confirm the patient’s MRN and their ICP physician(s). Next, the tablet is passed to the patient who enters the verification PIN (using the survey log-in feature in REDCap) based on their date of birth. Following successful verification, the patient may then complete their self-assessments, including the PHQ-9 [12], the Generalized Anxiety Disorder-7 [13], a substance use compliance screener, and a side-effects screener. Additional self-reports are completed at the initial and discharge appointments and include patient history, sociodemographics, the World Health Organization Disability Assessment Schedule 2 [14], the World Health Organization Quality of Life Instruments [15], and the Ontario Perception of Care [16] survey. In some use cases, the REDCap survey queue may be used to conditionally deploy certain assessments based on the responses provided in certain screener assessments. Once the patient has completed their assessments, a notification is emailed to the ICP physician with a link to a summary page in REDCap. This allows the physician to quickly review the results before and during the appointment without having to log into REDCap.

### Impact of Digitization

The digitization of the MDD-ICP MBC component contributed to a 4-fold adoption rate by physicians in the clinic compared with the paper-based pilot implementation phase (Table 1). Digitization resulted in an efficient and streamlined process that automatically calculated assessment scores and the ability for psychiatrists to access and review results before a patient’s appointment. This practice change, along with clinic-wide implementation, has contributed to the more than tripling of MDD-ICP patients seen by the clinic and has improved data quality. The use of Clinical REDCap ensures a standardized collection of assessments from patients at each visit and minimizes missing data. This systematic data collection method has resulted in the accumulation of high-quality data that may be used to inform quality improvement projects and to answer research questions with approval from the CAMH research ethics board.

**Table 1:** Overview of the impact and outcomes of Centre for Addiction and Mental Health major depressive disorder integrated care pathway digital transformation.

Measure	Digitization outcome	Impact
Physician adoption^a^	Contributed to a 4-fold increase in-clinic physician adoption rate Contributed to adoption by 20 physicians upon digitization compared with 5 physicians during the paper-based pilot implementation in the clinic	Increased capacity
Patient enrollment^a^	Contributed to a 4-fold increase in total patient enrollment on average per month After 6 months of digitization, an average of 49 patients enrolled monthly compared with 12 patients enrolled monthly during the paper-based pilot implementation	Increased capacity
Data entry time	Elimination of paper transcription by support staff Elimination of manual calculations	Increased workflow efficiency
Completeness	Major reduction of missing values and incomplete assessment packages Automatic date-stamping of all forms	Increased data quality
Standardization	3 new assessments submitted to the common data elements library for future deployment	Increased data quality

^a^Impact and outcome of clinic-wide major depressive disorder integrated care pathway implementation and digitization.

### User Acceptance

**Figure 5:**
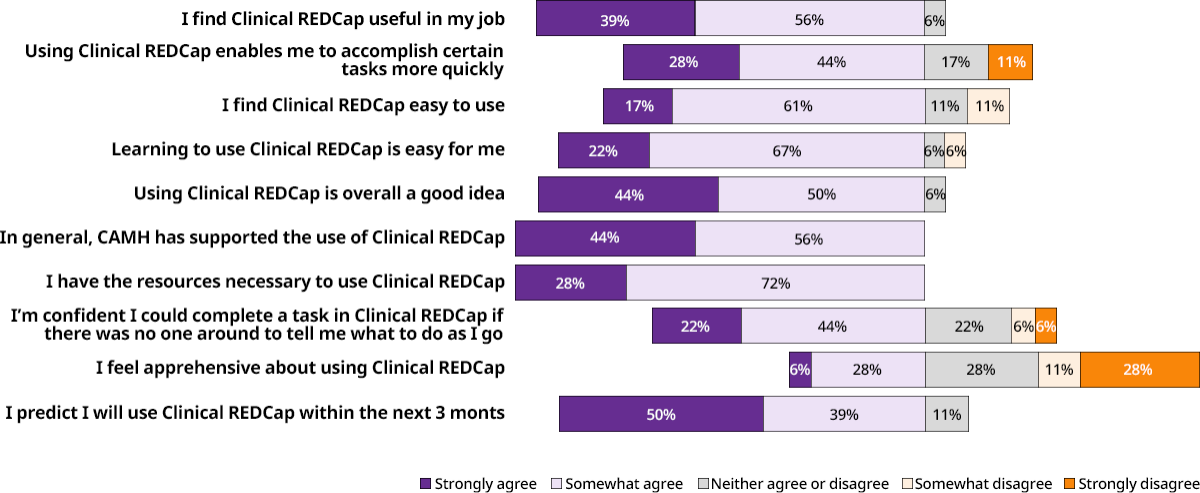
Clinical REDCap user acceptance questions and responses. CAMH: Centre for Addiction and Mental Health; REDCap: Research Electronic Data Capture.

All MDD-ICP clinical care providers were sent a 5-point Likert scale survey to assess their attitudes toward Clinical REDCap. A total of 18 responses were received, and Figure 5 shows the results of this survey.

Overall, respondents were satisfied with the system, with 94% (17/18) of respondents stating that they found Clinical REDCap useful in their job. Some apprehension was noted with ease of use, wherein 22% (4/18) to 33% (6/18) of respondents did not agree that the system was easy to use, that it allowed them to complete their work more quickly, or that they could complete a task without help. Despite these reservations, however, most respondents indicated that they planned to use the system within the next 3 months (use of the system is voluntary). From the free-text responses included in the survey, many respondents commented that electronic data capture presented major time savings compared with the previous paper-based entry method, especially in terms of the automatic calculation of scale scores. Conversely, many respondents noted that the interface could be more flexible and user-friendly with better tools for tracking patient trajectories.

## Discussion

### Principal Findings

Ensuring sustained high-quality data collection is critical for supporting actionable MBC and clinical decision making. REDCap has proven to be highly valuable for deploying complex self-assessment workflows in diverse clinical environments. A governance model was established consistent with the HIMSS 7 designated processes that ensure regulatory compliance and integrity of the health record. System integration between REDCap and Cerner established real-time data flow for completed self-assessments.

Almost all clinical care provider users of Clinical REDCap indicated that they perceived the system to be useful; however, the survey revealed some concerns with the perceived ease of use of Clinical REDCap. These concerns are worth noting given that a user’s motivation to use technology is higher if the system is perceived as easy to use [17,18]. However, despite these concerns, all survey respondents indicated that they felt supported by the institution and that they had the resources available to use the system.

Efforts are underway to streamline workflows and improve integration and reporting. Currently, customized summary reports on questionnaire results are generated by Clinical REDCap and sent to physicians to review ahead of the patient’s appointments for clinical decision making. This process has also had a positive impact on patient-physician communication and interaction during visits. However, the current presentation of self-report questionnaire results and EHR data focuses on point-of-care evaluation; the accessibility and presentation of complete treatment response trajectory over time would further enhance clinical decision support. To this end, enabling interactive data visualization methods (ie, dashboards) to convey knowledge about an individual’s treatment trajectory would provide health care providers a tool for rapidly processing and interpreting the patient’s chart and enhancing individualized clinical treatment decisions. Such a tool would free physicians from searching and filtering through the patient’s chart, which can be challenging in a time-pressured clinical setting. Thus, both Clinical REDCap and EHR dashboards will be codeveloped with physicians using human-centered design methodology and embedded within the EHR to ensure streamline access during patient appointments. For the MDD-ICP, this dashboard will feature self-report measures trended over time along with corresponding treatment such as medication.

One of the core features of REDCap is the ability to send survey invitations via email, thereby allowing respondents to complete self-assessments at their convenience using their own devices. With the recent pandemic and temporary suspension of in-person clinic visits, the demand for such functionality (along with other virtual care solutions) has never been greater. An approach to this use case using Clinical REDCap may be configuring external access only to REDCap survey pages while prohibiting project backend access. This approach would allow off-site patients to complete their self-assessments while simultaneously protecting the project data and setup pages on the internal network. Although this strategy may precipitate novel privacy concerns surrounding the use of unsecured email (eg, sending to the wrong email address or guardians having access to their dependent’s email account), this concern may be mitigated by using a patient verification PIN and other verification methods. Moreover, only blank self-assessment forms would be sent by email, and no sensitive health data or identifying information would be included. Consent language informing patients of these potential risks would be another key element in risk mitigation. A new process using this approach is currently being explored in several clinics that aim to send patients their self-assessment package 3 to 5 days before their appointment, followed by a reminder the day before. The self-assessment summary report can then be reviewed by the clinician or physician during a virtual appointment. It is anticipated that this new process will be able to leverage existing workflows and should not incur a significant increase in workload on the clinical staff compared with the original tablet-based approach.

The design of MBC pathways in Clinical REDCap will facilitate the rapid deployment of these pathways in other CAMH clinics. In addition, the scalable and extensible design of Clinical REDCap presents an opportunity for CAMH to partner with community sites to jointly implement MBC pathways in primary care settings. The use of assessments drawn from the common data elements library will enable standardized data collection practices consistent across the institution(s), thereby facilitating the accumulation of harmonized data that will allow clinicians and researchers to make meaningful comparisons over time and across otherwise independent clinical environments.

### Conclusions

REDCap has been successfully deployed for the structured collection of standardized self-assessments to coordinate and accelerate the implementation of MBC in clinical practice. Systems integration was achieved between REDCap and Cerner to automatically transfer PDF and discrete variables from completed assessments into the patient’s chart. This transfer maintained the integrity of the legal health record and enhanced clinician or physician decision-making ability. Thus, both a novel technical infrastructure and a comprehensive governance model aligned with institutional clinical committees were created to implement this new technology within the clinic. REDCap was demonstrated to be an effective tool for the implementation and expansion of MBC and ICPs by supporting an efficient and standardized process to deliver complex longitudinal bundles of assessments for the duration of a patient’s treatment and by delivering real-time accessible information to health care providers to inform clinical decisions. This digitization process and integration has been designed to be extensible to other organizations practicing MBC in mental health and other disciplines.

## Abbreviations

API: application programming interface
CAMH: Centre for Addiction and Mental Health
CSV: comma-separated values
DET: data entry trigger
EHR: electronic health record
HIMSS: Healthcare Information Management Systems Society
ICP: integrated care pathway
JSON: JavaScript Object Notation
MBC: measurement-based care
MDD-ICP: major depression disorder integrated care pathway
MRN: medical record number
PHQ-9: Patient Health Questionnaire-9
PIN: personal identification number
SQL: Structured Query Language
